# In vivo inhibition of cysteine proteinases delays the onset of growth of human pancreatic cancer explants

**DOI:** 10.1054/bjoc.1999.1021

**Published:** 2000-02

**Authors:** C J F Van Noorden, T G N Jonges, L C Meade-Tollin, R E Smith, A Koehler

**Affiliations:** Department of Cell Biology and Histology, Academic Medical Center, University of Amsterdam, Meibergdreef 15, Amsterdam, AZ, 1105, The Netherlands; Department of Pathology, Academic Hospital Utrecht, The Netherlands; Department of Surgery, University of Arizona College of Medicine, P.O. Box 245084, Tucson, AZ, 85724, USA; Enzyme Systems Products and Prototek, 486 Lindbergh Avenue, Livermore, CA, 94550, USA; Department of Cell Biology and Pathology, Biologische Anstalt in the Alfred Wegener Institute, Notkestrasse 85, Hamburg, D-22607, Germany

**Keywords:** proteinase, cysteine proteinases, proteinase inhibitor, pancreatic cancer, nude mice, in vivo

## Abstract

An animal model was used to study the effects of oral treatment with a small molecular selective inhibitor of cysteine proteinases, Z-Phe-Arg-fluoromethylketone (Z-Phe-Arg-FMK) on primary tumour development. Poorly differentiated rapidly growing and moderately differentiated slowly growing human pancreatic tumours were implanted in the neck of nude mice that were orally treated or not with the inhibitor. Growth rates of the tumours were determined during 38 days after implantation. The poorly differentiated tumours were not affected by treatment with the inhibitor. Development of the moderately differentiated tumours was inhibited significantly by Z-Phe-Arg-FMK treatment. Moreover, the amount of stroma was increased and the volume of cancer cells was reduced in the moderately differentiated tumours that had grown in the treated animals. Reduction in size of the tumours was not achieved by reduction in growth rate but in a delay of the onset of growth. It is concluded that cysteine proteinases play a transient role at the start of tumour development only when cancer cells are surrounded by stroma as was the case in the moderately differentiated but not in the poorly differentiated pancreatic tumours. However, this role of cysteine proteinases can easily be taken over by other proteinases. © 2000 Cancer Research Campaign


*British Journal of Cancer* (2000) **82**(4), 931–936
© 2000 Cancer Research Campaign
DOI: 10.1054/ bjoc.1999.1021, available online at http://www.idealibrary.com on IDEAL®

### In vivo inhibition of cysteine proteinases delays the onset of growth of human pancreatic cancer explants

**CJF Van Noorden[1], TGN Jonges[2], LC Meade-Tollin[3], RE Smith[4] and A Koehler[5]**

[1]Academic Medical Center, University of Amsterdam, Department of Cell Biology and Histology, Meibergdreef 15, 1105 AZ Amsterdam, The Netherlands; [2]Department of Pathology, Academic Hospital Utrecht, The Netherlands; [3]Department of Surgery, University of Arizona College of Medicine, P.O. Box 245084, Tucson, AZ 85724, USA; [4]Enzyme Systems Products and Prototek, 486 Lindbergh Avenue, Livermore, CA 94550, USA; [5]Department of Cell Biology and Pathology, Biologische Anstalt in the Alfred Wegener Institute, Notkestrasse 85, D-22607 Hamburg, Germany

**Summary** An animal model was used to study the effects of oral treatment with a small molecular selective inhibitor of cysteine proteinases, Z-Phe-Arg-fluoromethylketone (Z-Phe-Arg-FMK) on primary tumour development. Poorly differentiated rapidly growing and moderately differentiated slowly growing human pancreatic tumours were implanted in the neck of nude mice that were orally treated or not with the inhibitor. Growth rates of the tumours were determined during 38 days after implantation. The poorly differentiated tumours were not affected by treatment with the inhibitor. Development of the moderately differentiated tumours was inhibited significantly by Z-Phe-Arg-FMK treatment. Moreover, the amount of stroma was increased and the volume of cancer cells was reduced in the moderately differentiated tumours that had grown in the treated animals. Reduction in size of the tumours was not achieved by reduction in growth rate but in a delay of the onset of growth. It is concluded that cysteine proteinases play a transient role at the start of tumour development only when cancer cells are surrounded by stroma as was the case in the moderately differentiated but not in the poorly differentiated pancreatic tumours. However, this role of cysteine proteinases can easily be taken over by other proteinases. © 2000 Cancer Research Campaign

**Keywords**: proteinase; cysteine proteinases; proteinase inhibitor; pancreatic cancer; nude mice; in vivo

Cysteine proteinases are a class of proteinases that may be involved in invasion and metastasis of cancer cells in addition to members of the matrix metalloproteinase (MMP) family and urokinase-type plasminogen activator (uPA) (Reich et al, 1988; Mignatti and Rifkin, 1993; Sier et al, 1994; Elliott and Sloane, 1996; Van Noorden et al, 1998*a*). Cathepsin B is the most abundant representative of the cysteine proteinases in human cells and is normally present in lysosomes for protein degradation after phagocytosis or autophagy (Sheahan et al, 1989; Kirschke et al, 1995). Inhibition of cysteine proteinase activity significantly reduces intracellular protein degradation (Everts et al, 1985, 1999; Van Noorden and Everts, 1991). Under certain conditions, cathepsin B can be secreted. For example, it is secreted by macrophages during chronic inflammation (Reddy et al, 1995) and by chondrocytes during the acute phase of arthritis (Van Noorden et al, 1988, 1989). Sloane and co-workers established that secretion of cathepsin B and subsequent binding to the surface of cancer cells is significant for invasion and metastasis (Elliott and Sloane, 1996). We have postulated that expression of extracellular activity of cathepsin B is an early step in the proteolytic cascade involved in invasion and metastasis of cancer cells (Van Noorden et al, 1998*a*, 1998*b*). It activates uPA (Kobayashi et al, 1991), which in turn activates plasminogen to plasmin. Plasmin then can activate MMPs (Johnson et al, 1998). We have established that cathepsin B on the membrane of living rat colon cancer cells is active at physiological pH and that treatment of rats with a selective inhibitor of extracellular but not lysosomal cysteine proteinases, Mu-Phe-homoPhe-fluoromethylketone (Mu-Phe-homoPhe-FMK), suppresses the number of liver metastases of these cancer cells by 60% and their volume by 80% (Van Noorden et al, 1998*b*).

The present study was performed to further elucidate the role of extracellular cysteine proteinases in tumour progression. We have investigated the effects of systemic treatment of nude mice with the selective cysteine proteinase inhibitor, Z-Phe-Arg-FMK (Van Noorden et al, 1988), on the growth of human pancreatic adenocarcinoma explants as a model to study primary tumour development (Klöppel et al, 1988; Coen et al, 1992; Van Noorden et al, 1995). It has been shown that proteases also play a role in growth of xenografted tumours in nude mice (Noël et al, 1998). In our study, poorly differentiated rapidly growing and moderately differentiated slowly growing explants were compared. The explants were incubated subcutaneously (s.c.) in the necks of animals which had been either treated or not treated orally with Z-Phe-Arg-FMK for up to 38 days and the volume of the tumours was determined at regular intervals. After killing of the animals, the tumours were harvested and growth patterns and relative amounts of cancer cells, stroma and necrotic areas were determined. The poorly differentiated tumours were unaffected by treatment, whereas the moderately differentiated tumours were inhibited in growth by Z-Phe-Arg-FMK treatment during the first 4–9 days after implantation but not in later stages. More stroma and fewer cancer cells were present in the tumours grown in treated mice. This study demonstrates that cysteine proteinases play a role in early events of pancreatic tumour progression.

*Received 2 June 1999*
*Revised 19 August 1999*
*Accepted 23 August 1999*

*Correspondence to:* CJF Van Noorden

### MATERIALS AND METHODS

### Explants

Human pancreatic adenocarcinomas (PaCa 2 and PaCa 39) were grown as explants in athymic female Swiss-nu nude mice (IFFA Credo, Lyon, France) as described previously (Klöppel et al, 1988; Coen et al, 1992). PaCa 2 is poorly differentiated and grows rapidly, whereas PaCa 39 is moderately differentiated and grows slowly. Eight groups of ten mice were used in two similar experiments with 40 mice each. In each experiment, one group received PaCa 2 tumours and no Z-Phe-Arg-FMK, the second group received PaCa 2 tumours and Z-Phe-Arg-FMK, the third group received PaCa 39 tumours and no Z-Phe-Arg-FMK and the fourth group received PaCa 39 tumours and Z-Phe-Arg-FMK. On day 0, each mouse received two small pieces of either tumour (approximately $2 \times 2 \times 2$ $mm^3$) s.c. in the neck, one at each side. Forty mice were thus implanted with PaCa 2 tumours and 40 with PaCa 39 tumours. On day 38, the animals were killed under deep ether anaesthesia by cervical dislocation. Twenty animals with PaCa 2 tumours were killed on day 28 because the tumours became too large and started to interfere with the well-being of the animals. Animal welfare was maintained in accordance with the guidelines of the University of Amsterdam. At day 14 and subsequently every third day, the size of the tumours was determined with a sliding calipers and the tumour volume was estimated using the formula: Volume = $1/2 \times \text{Length} \times \text{Width}^2$ (Coen et al, 1992). After the animals were killed, tumours were removed and cut in half. All tumour fragments were frozen immediately in liquid nitrogen by placing them in Eppendorf vials (2 ml) which were capped with a vented lid and frozen slowly in liquid nitrogen (Van Noorden and Frederiks, 1992). Afterwards, the samples were stored at –80°C until further use.

### Treatment of mice with Z-Phe-Arg-FMK

Twenty mice with PaCa 2 and 20 mice with PaCa 39 tumours were treated orally with 20 mg Z-Phe-Arg-FMK per kg body weight dissolved in phosphate-buffered saline (PBS) and 5% dimethylformamide daily starting 1 week before administration of the tumours and continuing until sacrifice. Oral administration was performed directly into the stomach with a plastic tube attached to a syringe. Dosage and type of treatment were selected on the basis of previous experiments in which rats were treated with Z-Phe-Arg-FMK-related inhibitors of cysteine proteinases (Van Noorden et al, 1988; Ahmed et al, 1992; Esser et al, 1994). The other 40 animals were treated similarly with PBS and 5% dimethylformamide only. The $LD_{50}$ of Z-Phe-Arg-FMK is at least 20-fold higher than the dosages used in the present experiment (Ahmed et al, 1992; Esser et al, 1994).

### Tissue preparation

The unfixed frozen material was used for histochemical analysis as previously described (Vogels et al, 1993; Van Noorden et al, 1995). Serial cryostat sections (8 μm thick) from the surface of the halves of the tumours were cut on a motor-driven cryostat fitted with a retraction microtome at a cabinet temperature of –25°C. The sections were kept in the cryostat cabinet until used. Before use, the sections were allowed to dry for at least 5 min at 37°C or 10 min at room temperature. All incubations to demonstrate enzyme activity were performed on unfixed sections as described by Van Noorden and Frederiks (1992). For histochemical purposes, sections were fixed in 4% paraformaldehyde (Merck, Darmstadt, Germany) in PBS for 10 min at room temperature and washed three times in PBS for 2 min.

### Enzyme histochemical procedures

Cathepsin B activity was demonstrated with a fluorescence method as described previously (Van Noorden and Frederiks, 1992). Unfixed cryostat sections were incubated at room temperature on the stage of a fluorescence microscope (Leitz Orthoplan, Wetzlar, Germany) in a medium containing 100 mM phosphate buffer (pH 6.0), 1.3 mM EDTA (disodium salt), 1 mM dithiothreitol, 2.7 mM L-cysteine, 1 mM 2-hydroxy-5-nitrobenzaldehyde (Merck, Darmstadt, Germany) as coupling agent and 1 mM N-CBZ-Ala-Arg-Arg-4-methoxy-2-naphthylamide (Enzyme Systems Products, Livermore, CA, USA) as substrate for cathepsin B. Specificity of the reaction was verified by incubation in the presence of Z-Phe-Arg-FMK. Photomicrographs were taken at 15 min after the start of the incubation.

Lactate dehydrogenase activity was demonstrated in order to discriminate between viable and non-viable cells or necrosis (Frederiks et al, 1989). Sections were incubated for 4 min at 37°C in 100 mM phosphate buffer (pH 7.45), containing 18% polyvinyl alcohol (PVA) (weight average $M_r$, 70 000–100 000) (Sigma, St Louis, MO, USA), 150 mM sodium-L-lactate (Serva, Heidelberg, Germany), 3 mM $NAD^+$ (Boehringer, Mannheim, Germany), 0.45 mM 1-methoxyPMS (Sigma), 5 mM sodium azide and 5 mM tetranitroblue tetrazolium (Serva). Control reactions were carried out on consecutive sections with media lacking lactate. After incubation, sections were rinsed in PBS (60°C) and mounted in glycerol jelly.

### Histochemical procedures

Collagen was demonstrated with either Sirius red F3BA in a saturated aqueous solution of picric acid (James et al, 1986) or according to Shoobridge (1983).

In the first method, sections were stained for 1 h in 0.1% (w/v) Sirius red F3BA (Chroma, Stuttgart, Germany) in a saturated aqueous solution of picric acid (approx. 1.2% w/v) at pH 2.0. After staining, the slides were rinsed for 2 min in 0.01 N hydrochloric acid (HCl) to remove unbound dye. The dye bound to collagen is not removed at this pH (James et al, 1986).

The Shoobridge (1983) method was applied as follows: sections were washed in distilled water, treated with ferric alum (Chroma) for 10 min at room temperature, rinsed in distilled water and treated with Lillie's hemalum (Life Sciences, Zeist, The Netherlands) for 10 min at room temperature, washed in running tap water and immersed for 15–35 s in acid alcohol. After these steps, sections were rinsed for 10–15 min in running tap water and were immersed in prewarmed (50°C) distilled water. Sections were then treated with Naphthol Yellow S (Chroma) for 30 min at 50°C, and washed in running tap water (5 min). They were stained in tungsto-orange solution (Chroma) for 5 min at room temperature, rinsed in distilled water and stained with tungsto-acid fuchsin solution (Chroma) for 2 min at room temperature. Afterwards, sections were washed in distilled water. For the last staining step, sections were incubated in a tungsto-aniline blue solution (Chroma) for 5 min at room temperature and rinsed in distilled water.

© 2000 Cancer Research Campaign

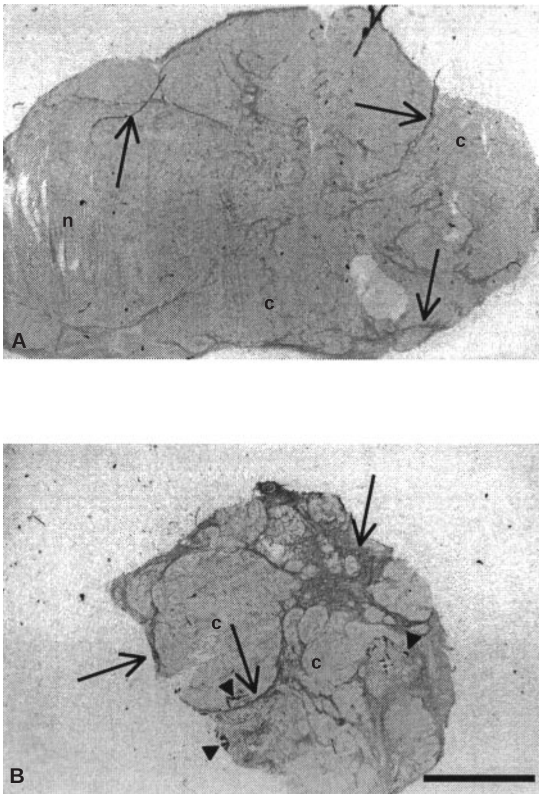

**Figure 1** Sections of poorly differentiated (**A**) and moderately differentiated (**B**) human pancreatic carcinoma explants that were grown in nude mice after staining with the pikrosirius red method to demonstrate collagen (arrows). Note that the amount of collagen in tumour B is much higher than in tumour A. n, necrosis; c, cancer cells; arrow heads, pieces of cartilage and bone (see Van Noorden et al, 1995). Bar = 5 mm

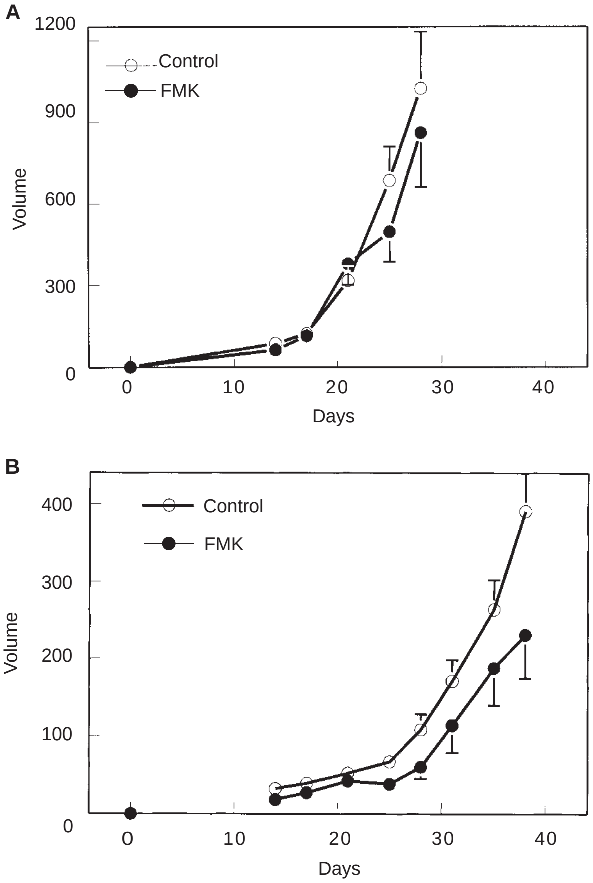

**Figure 2** Growth curves of rapidly growing poorly differentiated (**A**) and slowly growing moderately differentiated (**B**) human pancreatic carcinoma explants in nude mice. The mice were either not treated (○) or treated (●) orally with the selective cysteine proteinase inhibitor Z-Phe-Arg-FMK

### Morphometric analysis

From each tumour, consecutive cryostat sections were either incubated to demonstrate lactate dehydrogenase activity or stained with the picrosirius red method and used for morphometric analysis. Photomicrographs of the total area of each section (Figure 1) were taken with an AH-2 photomicroscope and a SPlan FL 2× objective (NA 0.08) (Olympus, Tokyo, Japan). Micrographs were printed at a final magnification of ×16. The micrographs were coded, randomized and subjected to a point-counting procedure using a double lattice testgrid with a 1:9 ratio (Weibel, 1963). In sections stained for lactate dehydrogenase activity, the area of the sections was determined (in $\mu m^2$) and the area density of viable and necrotic tissue was expressed as percentage of the total tissue area. In the consecutive sections stained for collagen, again the area of the tissue was determined (in $\mu m^2$) and the area density of stroma was expressed as percentage of the total tissue area.

### Statistical analysis

Data were analysed by Anova/Manova (Statistica; Statsoft, Tulsa, OK, USA) for variances, means (including a post hoc test according to Scheffé) and main effects. As level of significance, $P = 0.05$ was chosen.

### RESULTS

Rapidly and slowly growing tumours contained cathepsin B activity, both in cancer cells and stroma (data not shown). Treatment of mice with Z-Phe-Arg-FMK reduced activity (data not shown) in a comparable manner as reported previously in colon cancer metastases in rats (Van Noorden et al, 1998*b*).

Volumes of both rapidly and slowly growing tumours could be determined at first at approx. 14 days after implantation, irrespective of Z-Phe-Arg-FMK treatment. During the following 24 days, mean tumour volume of all groups of mice increased exponentially (Figure 2). In the first experiment, the average volume of the rapidly growing poorly differentiated tumours was approx. 1 $cm^3$ after 28 days of growth and that of slowly growing moderately differentiated tumours 5–10 times less (Figure 2). The animals with the rapidly growing tumours were killed then to avoid unnecessary suffering of the animals. In the second experiment, the rates of tumour growth were decreased so that an average size of 1 $cm^3$ of the PaCa 2 tumours was reached at 38 days (data not shown). Again the volume of the PaCa 39 tumours was 5–10 times less in that experiment.

### Rapidly growing poorly differentiated PaCa 2 tumours

Histochemical analysis of the poorly differentiated pancreatic tumours showed large areas of cancer cells surrounded by thin

© 2000 Cancer Research Campaign

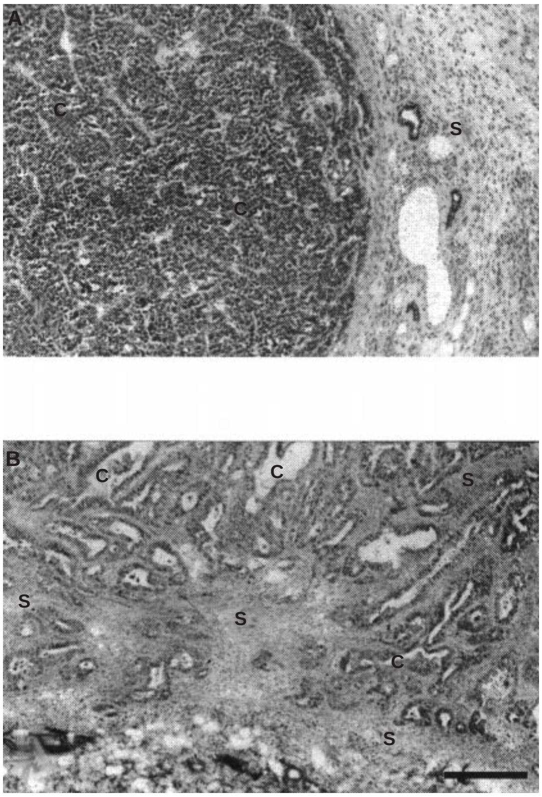

**Figure 3** Histology of poorly differentiated (**A**) and moderately differentiated (**B**) human pancreatic carcinoma explants that were grown in nude mice after Shoobridge staining. Note that the amount of stroma in tumour B is much higher than in tumour A and that the cancer cells in tumour B grow in duct-like structures. c, cancer cells; s, stroma. Bar = 100 μm

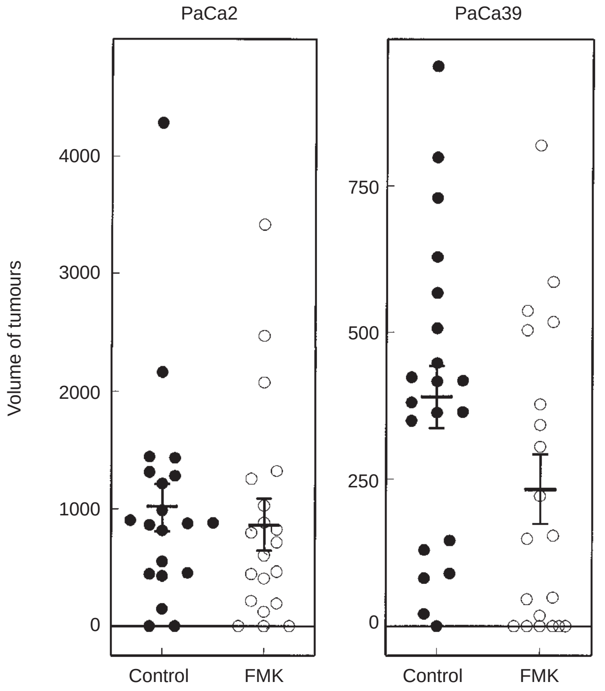

**Figure 4** End point volumes of rapidly growing poorly differentiated (PaCa 2) and slowly growing moderately differentiated (PaCa 39) human pancreatic tumours grown in nude mice that were not treated (●) or treated (○) orally with the selective cysteine proteinase inhibitor Z-Phe-Arg-FMK. Means ± s.e.m. of the volumes of the tumours are given as bars

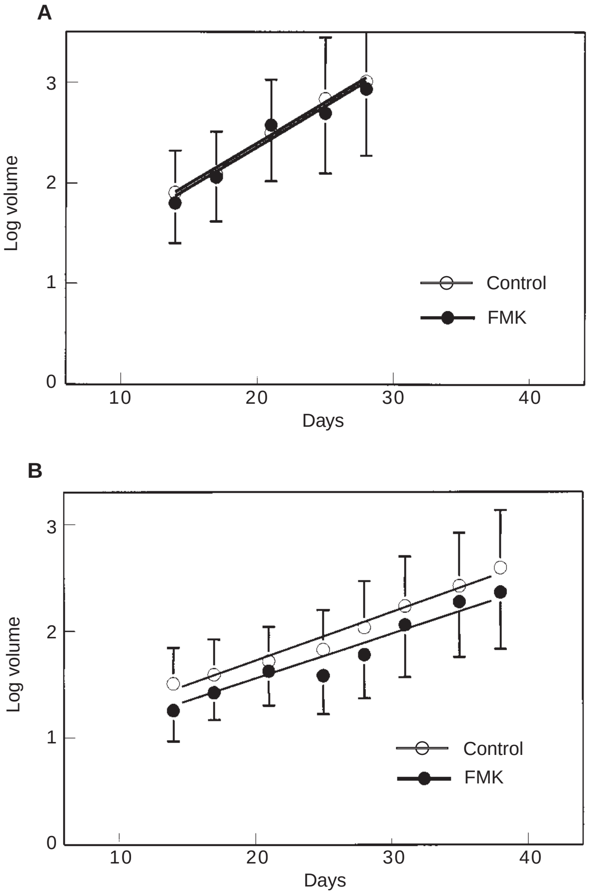

**Figure 5** Log transformation of volumes of rapidly growing poorly differentiated (**A**) and slowly growing moderately differentiated (**B**) human pancreatic tumours during development in nude mice that were either not treated (❍) or treated (●) orally with the selective cysteine proteinase inhibitor Z-Phe-Arg-FMK. The linear relationships are the best fits calculated on the basis of all tumour volumes determined at each time point during growth sheets of stroma (Figures 1A and 3A). Many necrotic areas were observed (Figure 1A). The respective amounts of cancer cells, necrotic areas and stroma were 49 ± 13% (mean percentage ± standard deviation (s.d.)), 28 ± 11% and 22 ± 8%. Z-Phe-Arg-FMK treatment did not significantly affect morphology of the tumours, growth patterns of the cancer cells or the respective percentage volumes that were occupied by cancer cells, necrosis and stroma. Treatment also did not affect growth rates of the tumours as measured by their volumes (Figures 2A and 4). Log transformed values of tumour volume versus period of development showed linear relationships that were similar for both untreated and Z-Phe-Arg-FMK-treated animals, in the first (Figure 5A) and the second experiment (data not shown). On the basis of these results, we concluded that systemic treatment with an inhibitor of cysteine proteinases does not affect growth of the poorly differentiated PaCa 2 explants.

### Slowly growing moderately differentiated PaCa 39 tumours

The moderately differentiated tumours showed duct-like arrangements of the cancer cells (Figure 3B). The duct-like structures

© 2000 Cancer Research Campaign were surrounded by stroma that was much more dispersed within the tumours (Figure 3B) than was the case in the poorly differentiated PaCa 2 tumours (Figure 3A). The percentage area of stroma was higher in PaCa 39 tumours than in PaCa 2 tumours (34 ± 8% vs 22 ± 8%). Necrosis was seldom found in PaCa 39 tumours. Z-Phe-Arg-FMK-treatment affected the amount of stroma in the tumours; it increased to a percentage area of 42 ± 12% at the cost of cancer cells. After 38 days of growth, untreated mice had tumours with an average volume of 391 ± 60 $mm^3$ (range 0–948 $mm^3$; Figures 2B and 4), whereas Z-Phe-Arg-FMK-treatment of the animals reduced the size to 231 ± 57 $mm^3$ (range 0–819 $mm^3$; Figures 2B and 4). The effect of Z-Phe-Arg-FMK was highly significant ($P < 0.001$). When the observation that Z-Phe-Arg-FMK-treatment increased the percentage area of stroma is taken into account, the cancer cell volume in the tumours decreased from 315 $mm^3$ to 176 $mm^3$ due to Z-Phe-Arg-FMK-treatment.

The plot of log transformed values of tumour volumes against period of time of growth shows significantly different linear relationships ($P < 0.001$) in Z-Phe-Arg-FMK-treated animals and untreated animals (Figure 5B). The lines are parallel indicating that the growth rate was not affected by treatment. However, the onset of tumour growth was delayed by Z-Phe-Arg-FMK-treatment. It can be calculated on the basis of Figure 5B, that the delay was 4 days in the first experiment, whereas in the second experiment the delay was calculated to be 9 days (data not shown). The relative amount of stroma in these tumours was again larger in the Z-Phe-Arg-FMK-treated animals as in the first experiment (data not shown).

On the basis of these data, we conclude that systemic Z-Phe-Arg-FMK-treatment of mice delays the onset of development of moderately differentiated tumours but does not affect the growth rate once the tumours start to grow.

### DISCUSSION

The present study of the effects of inhibition of cysteine proteinases on the growth of primary pancreatic adenocarcinomas shows that cysteine proteinases are involved in tumour progression but that their role is not essential. The most remarkable observation was that inhibition of cysteine proteinases delays the onset of growth of the moderately differentiated tumours but not the poorly differentiated tumours. Once the tumours start growing, growth rates are similar in treated and untreated animals. We explain these findings as follows. In the rapidly growing poorly differentiated tumours there are not many stromal elements around cancer cells in contrast with the slowly growing moderately differentiated tumours (Figure 3). Therefore, cancer cells in poorly differentiated tumours can proliferate without restraint right from the moment that the tumours have been implanted. In contrast, the cancer cells in the slowly growing tumours are surrounded by stroma, which is a barrier that has to be crossed before invasion and further development takes place. We have previously shown that the layer of stroma and connective tissue surrounding cancer cell nests is a far more important defence from host tissue against tumour progression than thus far has been anticipated (Griffini et al, 1997, 1998; Van Noorden et al, 1998*a*). Once the barrier is broken down, tumour development is unrestricted. It is that early phase of tumour progression in which cysteine proteinases seem to play a role. Our study demonstrates that the delay in onset of growth is 4–9 days in this tumour type. On the basis of these findings, we suggest that starting treatment with the cathepsin B inhibitor after tumours have been implanted is not likely to be successful in inhibiting tumour development. It may only have success in combination with inhibitors of other proteinases.

The significant increase in the amount of connective tissue in the slowly growing tumours due to treatment with Z-Phe-Arg-FMK can be explained by inhibition of breakdown of collagen by fibroblasts. Fibroblasts produce and degrade collagen. When the latter is inhibited by blocking cysteine proteinases, the balance between synthesis and degradation is disturbed, leading to increased amounts of collagen (Everts et al, 1985, 1994; Van Noorden and Everts, 1991).

Whether the role of cysteine proteinase activity is directly involved in breakdown of extracellular matrix components or plays a role in initiation of a proteolytic cascade via uPA activation that ultimately leads to MMP activity – as we have hypothesized previously (Van Noorden et al, 1998*b*) – cannot be decided on the basis of the present study. Nevertheless, the different effects of inhibition of cysteine proteinases on invasion of cancer cells surrounded by stroma compared to that of cancer cells that are not surrounded by connective tissue as shown here in the experimental animal model are in agreement with the finding that elevated cathepsin B activity is present in invasive fronts in human colorectal cancer and thus playing a role in invasion (Emmert-Buck et al, 1994; Hazen et al, submitted).

It should be realized that the role of cysteine proteinases is not essential for tumour development because other proteolytic systems can and do take over their role. In fact, this conclusion was also supported by our experiments with systemic administration of the selective inhibitor of extracellular cathepsin B, Mu-Phe-homoPhe-FMK, to rats that were given rat colon cancer cells that can metastasize in the liver. Tumour development was reduced but not completely inhibited (Van Noorden et al, 1998*b*). Therefore, the conclusion seems to be justified that inhibitors of cysteine proteinases per se are not sufficient for inhibition of tumour progression and metastasis. We currently investigate how combinations of inhibitors of the different proteases claimed to be involved in tumour progression such as aprotinin for uPA and batimastat for MMPs besides inhibitors of cysteine proteinases affect tumour development in animal models.

### ACKNOWLEDGEMENTS

The authors gratefully acknowledge the helpful suggestions of Ms Suzanne Pieper to interpret the data of tumour development, the design of the figures by Mr Jan Peeterse and the preparation of the manuscript by Mrs Trees MS Pierik.

### REFERENCES

Ahmed NK, Martin LA, Watts LM, Palmer J, Thornburg L, Prior J and Esser RE (1992) Peptidyl fluoromethyl ketones as inhibitors of cathepsin B. *Biochem Pharmacol* **44:** 1201–1207

Coen H, Kint H, Kuyper CF, Jonges GN, Dumarey N, Pauwels M, Klöppel G and Roels F (1992) Growth behavior of human pancreatic carcinoma xenografts correlates with nuclear features. *Cytometry* **13**: 775–781

Elliott E and Sloane BF (1996) The cysteine protease cathepsin B in cancer. *Perspect Drug Discov Design* **6**: 12–32

Emmert-Buck MR, Roth MJ, Zhuang Z, Campo E, Rozhin J, Sloane BF, Liotta LA and Stetler-Stevenson WG (1994) Increased gelatinase A (MMP-2) and cathepsin B activity in invasive tumor regions of human colon cancer samples. *Am J Pathol* **145**: 1285–1290

*© 2000 Cancer Research Campaign* 

Esser RE, Angelo RA, Murphey MD, Watts LM, Thornburg LP, Palmer JT, Talhouk JW and Smith RE (1994) Cysteine proteinase inhibitors decrease articular cartilage and bone destruction in chronic inflammatory arthritis. *Arthr Rheum* **37**: 236–247

Everts V, Beertsen W and Tigchelaar-Gutter W (1985) The digestion of phagocytosed collagen is inhibited by the proteinase inhibitors leupeptin and E-64. *Collagen Rel Res* **5**: 315–336

Everts V, Korper W, Niehof A, Jansen DC and Beertsen W (1994) Type VI collagen is phagocytosed by fibroblasts and digested in the lysosomal apparatus: involvement of collagenase, serine proteinases and lysosomal enzymes. *Matrix Biol* **14**: 665–676

Everts V, Korper W, Jansen DC, Steinfort J, Lammerse I, Heera S, Docherty AJP and Beertsen W (1999) Functional heterogeneity of osteoclasts: matrix metalloproteinases participate in osteoclastic resorption of calvarial bone but not in resorption of long bone. *FASEB J* **13**: 1219–1230

Frederiks WM, Marx F and Myagkaya GL (1989) The 'nothing dehydrogenase' reaction and the detection of ischaemic damage. *Histochem J* **21**: 565–573

Griffini P, Smorenburg SM, Verbeek FJ and Van Noorden CJF (1997) Three-dimensional reconstruction of colon carcinoma metastases in liver. *J Microsc* **187**: 12–21

Griffini P, Fehres O, Klieverik L, Vogels IMC, Tigchelaar W, Smorenburg SM and Van Noorden CJF (1998) Dietary Ω-3 polyunsaturated fatty acids promote colon carcinoma metastasis in rat liver. *Cancer Res* **58**: 3312–3319

James J, Bosch KS, Zuyderhoudt FMJ, Houtkooper JM and Van Gool J (1986) Histophotometric estimation of volume density of collagen as an indication of fibrosis in rat liver. *Histochemistry* **85**: 129–133

Johnsen M, Lund LR, Rømer J, Almholt K and Danø K (1998) Cancer invasion and tissue remodeling: common themes in proteolytic matrix degradation. *Curr Opin Cell Biol* **10**: 667–671

Kirschke H, Barrett AJ and Rawlings ND (1995) Cathepsin B (EC 3.4.22.1). In: *Protein Profile, Vol. 2: Lysosomal Cysteine Proteinases*, pp. 1588–1591. Academic Press: London

Klöppel G, Otto V, Baisch H and Von Bülow M (1988) Growth, stability and experimental treatment of human tumors after transplantation on thymus aplastic nude mice. *Verh Dtsch Ges Path* **72**: 1–61

Kobayashi H, Schmitt M, Goretzki L, Chucholowski N, Calvete J, Kramer M, Gunzler WA, Janicke F and Graeff H (1991) Cathepsin B efficiently activates the soluble and the tumor cell receptor-bound form of the proenzyme urokinase-type plasminogen activator (Pro-uPA). *J Biol Chem* **266**: 5147–5152

Mignatti P and Rifkin DB (1993) Biology and biochemistry of proteinases in tumor invasion. *Physiol Rev* **73**: 161–195

Noël A, Hajitou A, Hoir CL, Maquoi E, Baramova E, Lewalle J-M, Remacle A, Kebers F, Brown P, Calberg-Bacq C-M and Foidart J-M (1998) Inhibition of stromal matrix metalloproteases: effects on breast-tumor promotion by fibroblasts. *Int J Cancer* **76**: 267–273

Reddy VY, Zhang QY and Weiss SJ (1995) Pericellular mobilization of the tissue-destructive cysteine proteinases, cathepsins B, L, and S, by human monocyte-derived macrophages. *Proc Natl Acad Sci USA* **92**: 3849–3853

Reich R, Thompson EW, Iwamoto Y, Martin GR, Deason JR, Fuller GC and Miskin R (1988) Effects of inhibitors of plasminogen activator, serine proteinases, and collagenase IV on the invasion of basement membranes by metastatic cells. *Cancer Res* **48**: 3307–3312

Sheahan K, Shuja S and Murnane MJ (1989) Cysteine protease activities and tumor development in human colorectal carcinoma. *Cancer Res* **49**: 3809–3814

Shoobridge MPK (1983) A new principle in polychrome staining: a system of automated staining, complementary to hematoxylin and eosin, and usable as a research tool. *Stain Technol* **58**: 245–258

Sier CFM, Vloedgraven HJM, Ganesh S, Griffioen G, Quax PHA, Verheijen JH, Dooijewaard G, Welvaart K, Van de Velde CJH, Lamers CBHW and Verspaget HW (1994) Inactive urokinase and increased levels of its inhibitor type 1 in colorectal cancer liver metastasis. *Gastroenterology* **107**: 1449–1456

Van Noorden CJF and Everts V (1991) Selective inhibition of cysteine proteinases by Z-Phe-Ala-$CH_2F$ suppresses digestion of collagen by fibroblasts and osteoclasts. *Biochem Biophys Res Commun* **178**: 178–184

Van Noorden CJF and Frederiks WM (1992) *Enzyme Histochemistry. A Laboratory Manual of Current Methods*. BIOS: Oxford

Van Noorden CJF, Smith RE and Rasnick D (1988) Cysteine proteinase activity in arthritic rat knee joints and the effects of a selective systemic inhibitor, Z-Phe-Ala-$CH_2F$. *J Rheumatol* **15**: 1525–1535

Van Noorden CJF, Vogels IMC and Smith RE (1989) Localization and cytophotometric analysis of cathepsin B activity in unfixed and undecalcified cryostat sections of whole rat knee joints. *J Histochem Cytochem* **37**: 617–624

Van Noorden CJF, Jonges GN, Vogels IMC, Hoeben KA, Van Urk B and Everts V (1995) Ectopic mineralized cartilage formation in human undifferentiated pancreatic adenocarcinoma explants grown in nude mice. *Calcif Tissue Int* **56**: 145–153

Van Noorden CJF, Meade-Tollin LC and Bosman FT (1998*a*) Metastasis. *Am Sci* **86**: 130–141

Van Noorden CJF, Jonges GN, Van Marle J, Bissell ER, Griffini P, Jans M, Snel J and Smith RE (1998*b*) Heterogeneous suppression of experimentally induced colon cancer metastasis in rat liver lobes by inhibition of extracellular cathepsin B. *Clin Exp Metast* **16**: 159–167

Vogels IMC, Van Noorden CJF, Hoeben KA, Korper W, Jonges GN and Everts V (1993) Use of frozen biological material for combined light and electron microscopy. *Ultrastruct Pathol* **17**: 537–546

Weibel ER (1969) Stereological principles for morphometry in electron microscopic cytology. *Int Rev Cytol* **26**: 235–302

 *© 2000 Cancer Research Campaign*

## References

[PDF_00491] Ahmed N. K., Martin L. A., Watts L. M., Palmer J., Thornburg L., Prior J., Esser R. E. (1992). Peptidyl fluoromethyl ketones as inhibitors of cathepsin B. Implication for treatment of rheumatoid arthritis.. Biochem Pharmacol.

[PDF_00494] Coen H., Kint H., Kuyper C. F., Jonges G. N., Dumarey N., Pauwels M., Kloppel G., Roels F. (1992). Growth behavior of human pancreatic carcinoma xenograft correlates with nuclear features.. Cytometry.

[PDF_00499] Emmert-Buck M. R., Roth M. J., Zhuang Z., Campo E., Rozhin J., Sloane B. F., Liotta L. A., Stetler-Stevenson W. G. (1994). Increased gelatinase A (MMP-2) and cathepsin B activity in invasive tumor regions of human colon cancer samples.. Am J Pathol.

[PDF_00505] Esser R. E., Angelo R. A., Murphey M. D., Watts L. M., Thornburg L. P., Palmer J. T., Talhouk J. W., Smith R. E. (1994). Cysteine proteinase inhibitors decrease articular cartilage and bone destruction in chronic inflammatory arthritis.. Arthritis Rheum.

[PDF_00509] Everts V., Beertsen W., Tigchelaar-Gutter W. (1985). The digestion of phagocytosed collagen is inhibited by the proteinase inhibitors leupeptin and E-64.. Coll Relat Res.

[PDF_00516] Everts V., Korper W., Jansen D. C., Steinfort J., Lammerse I., Heera S., Docherty A. J., Beertsen W. (1999). Functional heterogeneity of osteoclasts: matrix metalloproteinases participate in osteoclastic resorption of calvarial bone but not in resorption of long bone.. FASEB J.

[PDF_00512] Everts V., Korper W., Niehof A., Jansen I., Beertsen W. (1995). Type VI collagen is phagocytosed by fibroblasts and digested in the lysosomal apparatus: involvement of collagenase, serine proteinases and lysosomal enzymes.. Matrix Biol.

[PDF_00520] Frederiks W. M., Marx F., Myagkaya G. L. (1989). The 'nothing dehydrogenase' reaction and the detection of ischaemic damage.. Histochem J.

[PDF_00525] Griffini P., Fehres O., Klieverik L., Vogels I. M., Tigchelaar W., Smorenburg S. M., Van Noorden C. J. (1998). Dietary omega-3 polyunsaturated fatty acids promote colon carcinoma metastasis in rat liver.. Cancer Res.

[PDF_00522] Griffini P., Smorenburg S. M., Verbeek F. J., van Noorden C. J. (1997). Three-dimensional reconstruction of colon carcinoma metastases in liver.. J Microsc.

[PDF_00528] James J., Bosch K. S., Zuyderhoudt F. M., Houtkooper J. M., van Gool J. (1986). Histophotometric estimation of volume density of collagen as an indication of fibrosis in rat liver.. Histochemistry.

[PDF_00531] Johnsen M., Lund L. R., Rømer J., Almholt K., Danø K. (1998). Cancer invasion and tissue remodeling: common themes in proteolytic matrix degradation.. Curr Opin Cell Biol.

[PDF_00540] Kobayashi H., Schmitt M., Goretzki L., Chucholowski N., Calvete J., Kramer M., Günzler W. A., Jänicke F., Graeff H. (1991). Cathepsin B efficiently activates the soluble and the tumor cell receptor-bound form of the proenzyme urokinase-type plasminogen activator (Pro-uPA).. J Biol Chem.

[PDF_00544] Mignatti P., Rifkin D. B. (1993). Biology and biochemistry of proteinases in tumor invasion.. Physiol Rev.

[PDF_00537] Mölling K. (1988). Onkogene und Zelldifferenzierung.. Verh Dtsch Ges Pathol.

[PDF_00546] Noël A., Hajitou A., L'Hoir C., Maquoi E., Baramova E., Lewalle J. M., Remacle A., Kebers F., Brown P., Calberg-Bacq C. M. (1998). Inhibition of stromal matrix metalloproteases: effects on breast-tumor promotion by fibroblasts.. Int J Cancer.

[PDF_00550] Reddy V. Y., Zhang Q. Y., Weiss S. J. (1995). Pericellular mobilization of the tissue-destructive cysteine proteinases, cathepsins B, L, and S, by human monocyte-derived macrophages.. Proc Natl Acad Sci U S A.

[PDF_00553] Reich R., Thompson E. W., Iwamoto Y., Martin G. R., Deason J. R., Fuller G. C., Miskin R. (1988). Effects of inhibitors of plasminogen activator, serine proteinases, and collagenase IV on the invasion of basement membranes by metastatic cells.. Cancer Res.

[PDF_00557] Sheahan K., Shuja S., Murnane M. J. (1989). Cysteine protease activities and tumor development in human colorectal carcinoma.. Cancer Res.

[PDF_00560] Shoobridge M. P. (1983). A new principle in polychrome staining: a system of automated staining, complementary to hematoxylin and eosin, and usable as a research tool.. Stain Technol.

[PDF_00563] Sier C. F., Vloedgraven H. J., Ganesh S., Griffioen G., Quax P. H., Verheijen J. H., Dooijewaard G., Welvaart K., van de Velde C. J., Lamers C. B. (1994). Inactive urokinase and increased levels of its inhibitor type 1 in colorectal cancer liver metastasis.. Gastroenterology.

[PDF_00582] Van Noorden C. J., Jonges G. N., Vogels I. M., Hoeben K. A., Van Urk B., Everts V. (1995). Ectopic mineralized cartilage formation in human undifferentiated pancreatic adenocarcinoma explants grown in nude mice.. Calcif Tissue Int.

[PDF_00588] Van Noorden C. J., Jonges T. G., Van Marle J., Bissell E. R., Griffini P., Jans M., Snel J., Smith R. E. (1998). Heterogeneous suppression of experimentally induced colon cancer metastasis in rat liver lobes by inhibition of extracellular cathepsin B.. Clin Exp Metastasis.

[PDF_00574] Van Noorden C. J., Smith R. E., Rasnick D. (1988). Cysteine proteinase activity in arthritic rat knee joints and the effects of a selective systemic inhibitor, Z-Phe-AlaCH2F.. J Rheumatol.

[PDF_00578] Van Noorden C. J., Vogels I. M., Smith R. E. (1989). Localization and cytophotometric analysis of cathepsin B activity in unfixed and undecalcified cryostat sections of whole rat knee joints.. J Histochem Cytochem.

[PDF_00592] Vogels I. M., Van Noorden C. J., Hoeben K. A., Korper W., Jonges G. N., Everts V. (1993). Use of frozen biologic material for combined light and electron microscopy.. Ultrastruct Pathol.

[PDF_00595] Weibel E. R. (1969). Stereological principles for morphometry in electron microscopic cytology.. Int Rev Cytol.

[PDF_00568] van Noorden C. J., Everts V. (1991). Selective inhibition of cysteine proteinases by Z-Phe-AlaCH2F suppresses digestion of collagen by fibroblasts and osteoclasts.. Biochem Biophys Res Commun.

